# Associations of lipid profiles with insulin resistance and β cell function in adults with normal glucose tolerance and different categories of impaired glucose regulation

**DOI:** 10.1371/journal.pone.0172221

**Published:** 2017-02-15

**Authors:** Shuang Zheng, Hua Xu, Huan Zhou, Xingxing Ren, Tingting Han, Yawen Chen, Huiying Qiu, Peihong Wu, Jun Zheng, Lihua Wang, Wei Liu, Yaomin Hu

**Affiliations:** Department of Endocrinology, Renji Hospital, School of Medicine, Shanghai Jiaotong University, Shanghai, China; Johns Hopkins University Bloomberg School of Public Health, UNITED STATES

## Abstract

**Aims:**

To investigate the associations of dyslipidemia with insulin resistance and β cell function in individuals with normal glucose tolerance (NGT) and different categories of impaired glucose regulation (IGR).

**Methods:**

544 subjects (365 with dyslipidemia and/or IGR and 179 with normal lipid and glucose tolerance) were enrolled in the study. All subjects underwent oral glucose tolerance test (OGTT). HOMA-IR was used to evaluate insulin sensitivity. Disposition index (DI) was used to evaluate β cell function. Multiple linear regression analysis was performed to assess correlations among lipid profiles, insulin resistance and β cell function.

**Results:**

Among subjects with NGT, those with dyslipidemia had higher level of HOMA-IR but lower level of DI. While among subjects with different categories of IGR, those with dyslipidemia and CGI had significantly decreased DI. No obvious differences of insulin resistance or β cell function were found in IFG or IGT subjects with or without dyslipidemia. TG and HDL-C were correlated with HOMA-IR (β = 0.79, p <0.001; β = -0.38, p = 0.027, respectively, compared with subjects in the low level groups). Moreover, TG and TC were negatively correlated with DI (β = -2.17, p = 0.013; β = -2.01, p = 0.034 respectively, compared with subjects in the low level groups) after adjusting for confounding parameters.

**Conclusions:**

Dyslipidemia induces insulin resistance and impaired β cell response to insulin resistance in individuals with NGT. Furthermore, dyslipidemia diminishes β cell function in subjects with CGI. TG and HDL-C were correlated with insulin resistance, and TG, TC were negatively correlated with β cell response to insulin resistance in non-diabetic individuals.

## Introduction

Type 2 diabetes mellitus (T2DM), as one of the most common chronic diseases, causes serious morbidity and mortality and also imposes a heavy economic burden in the world [1]. Therefore, it is necessary to identify high-risk individuals and establish a useful primary prevention program for T2DM.

T2DM is frequently accompanied with dyslipidemia, which is known as an independent risk factor of T2DM [2]. However, one question is still partially unraveled: how does dyslipidemia affect diabetes development? In order to answer this question, we should investigate the role of dyslipidemia in β cells function in an early stage, namely non-diabetic subjects. Previous studies have demonstrated the phenomenon that impaired pancreatic β cells function caused by dyslipidemia precedes the manifestation of T2DM [3–7]. Additionally, the atherogenic lipid pattern is not only apparent in diabetic individuals but also in individuals with NGT [8–10]. However, few studies have clearly clarified the effects of dyslipidemia on insulin resistance and β cell function in subjects with NGT and different categories of IGR, including impaired fasting glucose (IFG), impaired glucose tolerance (IGT) and combined glucose intolerance (CGI). Besides, the associations of different lipid indices with insulin resistance and β cell function need further investigation in those non-diabetic individuals, which could provide more evidence of the utilization of these lipid indices as potential clinical tools for screening of high-risk individuals.

Thus, the aims of the study were to investigate the effects of dyslipidemia on insulin resistance and β cell function in non-diabetic individuals including those with NGT and different categories of IGR. Subsequently, the correlations of different lipid indices with insulin resistance and β cell function were further explored among those individuals.

## Subjects and methods

### Subjects

All subjects in this cross-sectional study were recruited from the outpatient clinic of Renji Hospital, School of Medicine, Shanghai Jiaotong University from January 2008 to December 2014. Initially, a total of 724 self-reported non-diabetic subjects aged from 18 to 80 years old were invited to the study and finished structured questionnaires at the first visit. Next, 146 subjects were excluded relying on the exclusion criteria, including pregnancy, hyperthyroidism or hypothyroidism, serious diseases like renal or hepatic insufficiency, heart disease, cancer, current history of cigarette smoking or alcohol drinking, taking regular medication for diabetes and/or dyslipidemia. Then, 34 subjects with newly diagnosed diabetes according to the results of oral glucose tolerance test (OGTT) in our study were further excluded. Finally, a total of 544 subjects were enrolled to the recent study. Of these individuals, 365 were patients with dyslipidemia and/or IGR, and 179 were subjects with normal lipid and glucose tolerance.

According to the 1999 World Health Organization criteria [11], NGT was defined as fasting glucose <6.1mmol/L and 2-h glucose < 7.8mmol/L, IFG was defined as fasting glucose between 6.1mmol/L and 7.0mmol/L and 2-h glucose < 7.8mmol/L, IGT was defined as fasting glucose <6.1mmol/L and 2-h glucose between 7.8mmol/L and 11.1mmol/L, CGI was defined as fasting glucose between 6.1mmol/L and 7.0mmol/L and 2-h glucose between 7.8mmol/L and 11.1mmol/L. Dyslipidemia was considered when triglyceride (TG) levels were ≥ 1.7mmol/L and/or total cholesterol (TC) levels ≥ 5.2 mmol/L and/or high density lipoprotein-cholesterol (HDL-C) levels <1.04 mmol/L and/or low density lipoprotein-cholesterol (LDL-C) levels ≥ 3.5mmol/L.

Subjects were categorized into normal lipid subgroups (NL) and dyslipidemia subgroups (DL) in both NGT and IGR groups. Additionally, subjects with IGR were further subdivided into IFG, IGT and CGI subgroups.

The study was carried out in accordance with the declaration of Helsinki and the study protocol was approved by the Ethical Committee of Renji Hospital, School of Medicine, Shanghai Jiaotong University, Shanghai, China. Before the participation, written informed consents were obtained from participants.

### Data collection

#### Anthropometry measurements

Body height, weight, waist circumference (WC), hip circumference and blood pressure (BP) were measured by trained survey personnel. Body height was measured once with a portable height scale to the nearest 0.1 cm. Weight was measured using a platform digital scale to the nearest 0.1kg. Both height and weight measurements were taken in light clothing without shoes. WC was measured as the midpoint circumference between the iliac crest and the lowest rib. Hip circumference was recorded as the largest gluteal circumference. Circumference measurements were taken twice by a single observer and the mean value was reported. Blood pressure was measured twice in each subject on the right arm after five minutes resting in a sitting position, and the mean of two measurements was considered.

#### Laboratory analysis

After a more than 8-hour overnight fast, plasma sample was taken from each subject for the measurement of fasting plasma glucose, fasting insulin and lipids levels. Besides, the blood samples were also obtained at 30, 60, 120, and 180 minutes after 75 g glucose load (a dose of the standard OGTT) to measure the concentration of glucose and insulin.

Glucose and lipid levels were measured using fully automatic biochemistry analyzer (Hitachi 7600–110 and Hitachi 7020, respectively, Hitachi Co. Tokyo, Japan); insulin concentration was determined by immunoradiometric assay kit (Dainabot, Tokyo, Japan); Glycated hemoglobin A1c (HbA1c) level was measured by a high-performance liquid chromatography.

#### Calculation

Body mass index (BMI) was defined as the body weight (kg) divided by the square of body height (m^2^). Waist to hip ratio (WHR) was calculated as waist circumference (cm) divided by hip circumference (cm). Index of insulin resistance (or sensitivity) was calculated from the OGTT data: homeostasis model assessment for insulin resistance (HOMA-IR) [HOMA-IR = fasting insulin (μU/ml) × fasting glucose (mmol/L) /22.5]. Disposition index (DI) was used to evaluate β cell function [DI = IGI/HOMA-IR, IGI = insulin in 30 min-fasting insulin (μU/ml)/ Glucose in 30min-fasting glucose (mmol/L)].

#### Statistical analysis

All statistical analyses were conducted utilizing SPSS Version 17.0 (SPSS Inc., Chicago, IL, USA). Normality of data was tested applying the Kolmogorov-Smirnov test. Data were expressed as mean ± standard deviation for normally distributed continuous variables and as median (Interquartile range 25–75%) for skewed variables. Group based differences were compared by analysis of variance (ANOVA) following SNK post hoc pairwise comparison for normality distributed data and Kruskal-Wallis H test following Mann–Whitney U test for skewed data. Adjusted means were calculated and compared with general linear models. Univariate liner regression analysis was applied to investigate the associations of demographic and clinical characteristics with insulin resistance and β cell function. Multiple liner regression analysis was further conducted to determine the correlations of different lipid indices with insulin resistance and β cell function after controlling potential confounding factors. In this part, all lipid indices were categorized into low level and high level groups according to the clinical diagnostic criteria. And for each indicator, the coefficient β and 95% CIs of the high level group was calculated and compared using the low level group as the reference. Statistical significance was defined as p value <0.05.

## Results

### Basic characteristics

Anthropometric and metabolic characteristics of the study groups were presented in Table 1. Genders and age were equally represented across different groups. Subjects with dyslipidemia and/or IGR had significantly higher systolic blood pressure, diastolic blood pressure, BMI and WHR than those with normal lipid and glucose regulation. Moreover, IGR groups showed higher BMI than NGT groups.

**Table 1:** Characteristics of the subjects according to glucose tolerance and serum lipid status.

Clinical and metabolic data	NGT		IGR	
	NL	DL	NL	DL
Number	179	108	70	187
Age(years)	52.0 (38.0,58.0)	49.0 (38.0,58.8)	51.0 (42.0,55.3)	52.0 (48.0,56.0)
Sex (M/F)	82/97	50/58	28/42	80/107
SBP(mmHg)	111.0 (102.0,121.0)	115.0 (110.0,127.5)^1^	121.0 (109.8,134.3)^1^	118.0 (110.0,131.0)^1^
DBP(mmHg)	72.0 (63.0,80.0)	77.0 (71.3,83.8)^1^	77.5 (70.0,81.0)^1^	79.0 (71.0,85.0)^1^
BMI(kg/m^2^)	22.19±0.22	25.77±0.28^1^	24.13±0.26^1^^,^^2^	24.49±0.29^1^^,^^2^
WHR	0.84±0.01	0.90±0.01^1^	0.89±0.02^1^	0.91±0.01^1^
FPG (mmol/L)	5.09±0.04	5.39±0.05^1^	5.62±0.07^1^^,^^2^	5.99±0.04^1^^,^^2^^,^^3^
2h PG(mmol/L)	5.97±0.07	6.12±0.15	8.38±0.13^1^^,^^2^	8.63±0.09^1^^,^^2^
FINS (μU/ml)	8.25 (5.78,11.30)	11.43 (8.54,17.02)^1^	9.77 (8.21,15.30)^1^	10.37 (7.73,15.03)^1^
2hINS(μU/ml)	36.12 (25.10,50.47)	39.75 (24.35,52.83)	50.74 (34.77,92.22)^1^^,^^2^	53.62 (31.01,81.44)^1^^,^^2^
HbA1c (%)	5.5 (5.3,5.8)	5.7 (5.2,5.9)	5.6 (5.3,6.0)	5.8 (5.5,6.2)^1^^,^^2^^,^^3^
TG(mmol/L)	0.92 (0.66,1.14)	1.79 (1.36,2.09)^1^	1.02 (0.76,1.32)^1^^,^^2^	1.79 (1.49,2.24)^1^^,^^3^
TC(mmol/L)	4.48 (3.86,4.82)	5.28 (4.58,5.53)^1^	4.56 (3.87,4.87)^2^	5.36 (4.77,5.82)^1^^,^^2^^,^^3^
HDL-C(mmol/L)	1.52 (1.32,1.76)	1.32 (1.08,1.62)^1^	1.34 (1.16,1.56)^1^	1.19 (1.00,1.54)^1^^,^^2^^,^^3^
LDL-C(mmol/L)	2.59 (2.19,2.92)	3.17 (2.61,3.56)^1^	2.72 (2.04,3.09)^1^^,^^2^	3.37 (3.04,3.71)^1^^,^^2^^,^^3^

Data were expressed as mean ± standard deviation for normal distribution and as median (Interquartile range 25–75%) for skewed variables.

NGT: normal glucose tolerance; IGR: impaired glucose tolerance; NL: normolipidemia; DL: dyslipidemia; SBP: systolic blood pressure; DBP: diastolic blood pressure; BMI: body mass index; WHR: waist to hip ratio; FPG: fasting plasma glucose; 2hPG: 2 hour postload plasma glucose; FINS: fasting serum insulin; 2hINS:2 hour postload serum insulin; HbA1c: glycated hemoglobin A1c; TG: triglycerides; TC: total cholesterol; HDL-C: high density lipoprotein cholesterol; LDL-C: low density lipoprotein cholesterol.

^1^P < 0.05 versus NGT/NL group,

^2^P < 0.05 versus NGT/DL group,

^3^P < 0.05 versus IGR/NL group.

Subjects with dyslipidemia had higher TG, TC and LDL-C levels and lower HDL-C concentrations than those with normal lipid. Notably, IGR/DL group had the worst aforementioned lipid profiles in these groups.

Individuals with dyslipidemia and/or IGR had higher levels of fasting glucose and fasting insulin than NGT/NL group. IGR groups had higher 2-h postload glucose and insulin than NGT groups. Compared with other groups, IGR/DL group had the highest level of HbA1c.

### Effects of dyslipidemia on insulin sensitivity and β cell function

**Fig 1:**
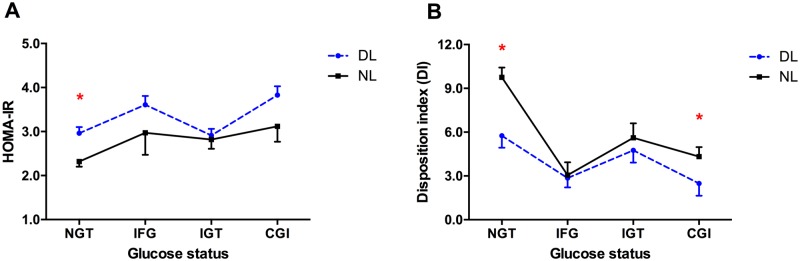
Insulin resistance and β cell function evaluation in non-diabetic subjects with or without dyslipidemia. HOMA-IR: homeostasis model assessment for insulin resistance, DI: disposition index, NL: normal lipid, DL: dyslipidemia, NGT: normal glucose tolerance, IFG: impaired fasting glucose, IGT: impaired glucose tolerance, CGI: combined glucose intolerance. *P < 0.05 versus normal lipid (NL) group. Data were expressed as mean ± SD, which were calculated after adjusting for age, sex, SBP, DBP, BMI and WHR.

Insulin resistance was evaluated by HOMA-IR, which was markedly elevated in patients with dyslipidemia in NGT group (Fig 1A). In addition, patients with dyslipidemia had lower levels of glucose disposition index (DI), especially in NGT and CGI groups (Fig 1B). Thus, subjects with NGT and dyslipidemia had higher insulin resistance and lower β cell function than those with normolipidemia. While subjects with CGI and dyslipidemia had lower β cell function than with normolipidemia.

### Associations among different lipid indices, insulin sensitivity and β cell function

Correlations between clinical variables and indices of insulin resistance and β cell function, namely HOMA-IR and DI, were evaluated with univariate correlation analysis (Table 2). Age and HDL-C were found to have negative correlations, and SBP, DBP, BMI, WHR and other lipid profiles had positive correlations with HOMA-IR. The SBP, DBP, BMI, WHR and lipid profiles (excluding HDL-C) were negatively correlated with DI.

**Table 2:** Univariate correlation between HOMA-IR/DI and various parameters.

	HOMA-IR		DI	
	*β*	*P value*	*β*	*P value*
Age	-0.168	<0.001	-0.041	0.356
Sex	-0.026	0.552	-0.084	0.058
SBP	0.130	0.002	-0.143	0.001
DBP	0.264	<0.001	-0.134	0.002
BMI	0.392	<0.001	-0.114	0.009
WHR	0.114	0.008	-0.165	<0.001
TG	0.460	<0.001	-0.316	<0.001
TC	0.194	<0.001	-0.245	<0.001
HDL-C	-0.292	<0.001	0.079	0.072
LDL-C	0.185	<0.001	-0.203	<0.001

HOMA-IR: homeostatic model assessment of insulin resistance; DI: disposition index; SBP: systolic blood pressure; DBP: diastolic blood pressure; BMI: body mass index; WHR: waist to hip ratio; TG: triglyceride; TC: total cholesterol; HDL-C: high-density lipoprotein cholesterol; LDL-C: low-density lipoprotein cholesterol.

Coefficients (β) and P values were calculated using the liner regression models.

Subsequently, multiple linear regression analysis was implemented to determine the influence of lipid profiles on HOMA-IR and DI. The results revealed that after adjusting for confounding factors, TG and HDL-C were correlated with HOMA-IR [For TG, adjusted β (95%CIs) of high level group was 0.79 (0.52, 1.06), p <0.001; for HDL-C, adjusted β (95%CIs) of high level group was -0.38 (-0.71, -0.05), p = 0.027] (Table 3). Moreover, TG and TC were negatively correlated with DI after adjusting for SBP, DBP, BMI and WHR [For TG, adjusted β (95%CIs) of high level group was -2.17 (-3.88, -0.46), p = 0.013; for TC, adjusted β (95%CIs) of high level group was -2.01 (-3.86, -0.15), p = 0.034] (Table 4).

**Table 3:** Multiple regression analysis of lipid indices associated with HOMA-IR.

	NGT				IGR				Total			
	Crude	P-value	Adjusted*	P-value	Crude	P-value	Adjusted*	P-value	Crude	P-value	Adjusted*	P-value
	β (95%CIs)		β (95%CIs)		β (95%CIs)		β (95%CIs)		β (95%CIs)		β (95%CIs)	
**TG**												
**<1.7**	0 (ref.)		0 (ref.)		0 (ref.)		0 (ref.)		0 (ref.)		0 (ref.)	
**≥1.7**	0.87	<0.001	0.70	<0.001	1.20	<0.001	0.86	<0.001	1.02	<0.001	0.79	<0.001
	(0.50,1.23)		(0.35,1.04)		(0.75,1.65)		(0.44,1.28)		(0.73,1.31)		(0.52,1.06)	
**TC**												
**<5.2**	0 (ref.)		0 (ref.)		0 (ref.)		0 (ref.)		0 (ref.)		0 (ref.)	
**≥5.2**	0.09	NS	-0.16	NS	0.82	<0.001	0.61	0.002	0.46	0.002	0.25	NS
	(-0.38,0.56)		(-0.67,0.35)		(0.45,0.18)		(0.22,1.00)		(0.17,0.75)		(-0.07,0.57)	
**HDL-C**												
**<1.04**	0 (ref.)		0 (ref.)		0 (ref.)		0 (ref.)		0 (ref.)		0 (ref.)	
**≥1.04**	-0.60	<0.001	-0.39	0.017	-0.53	0.009	-0.37	0.033	-0.59	<0.001	-0.38	0.027
	(-0.94,-0.26)		(-0.71,-0.07)		(-0.91,-0.14)		(-0.71,-0.03)		(-0.85,-0.33)		(-0.71,-0.05)	
**LDL-C**												
**<3.5**	0 (ref.)		0 (ref.)		0 (ref.)		0 (ref.)		0 (ref.)		0 (ref.)	
**≥3.5**	-0.23	NS	-0.23	NS	-0.47	NS	-0.08	NS	-0.38	0.026	-0.21	NS
	(-0.65,0.18)		(-0.63,0.17)		(-1.00,0.07)		(-0.58,0.41)		(-0.72,-0.05)		(-0.52,0.09)	

NGT: normal glucose tolerance; IGR: impaired glucose regulation; HOMA-IR: homeostatic model assessment of insulin resistance; TG: triglyceride; TC: total cholesterol; HDL-C: high-density lipoprotein cholesterol; LDL-C: low-density lipoprotein cholesterol.

*Analysis was conducted after adjusting for age, SBP, DBP, BMI, WHR.

**Table 4:** Multiple regression analysis of lipid indices associated with DI.

	NGT				IGR				Total			
	Crude	P-value	Adjusted*	P-value	Crude	P-value	Adjusted*	P-value	Crude	P-value	Adjusted*	P-value
	β (95%CIs)		β (95%CIs)		β (95%CIs)		β (95%CIs)		β (95%CIs)		β (95%CIs)	
**TG**												
**<1.7**	0 (ref.)		0 (ref.)		0 (ref.)		0 (ref.)		0 (ref.)		0 (ref.)	
**≥1.7**	-3.17	NS	-2.57	NS	-1.73	<0.001	-1.72	<0.001	-2.41	0.005	-2.17	0.013
	(-6.52,0.18)		(-6.03,0.89)		(-2.56,-0.89)		(-2.56,-0.88)		(-4.07,-0.74)		(-3.88,-0.46)	
**TC**												
**<5.2**	0 (ref.)		0 (ref.)		0 (ref.)		0 (ref.)		0 (ref.)		0 (ref.)	
**≥5.2**	-0.23	NS	-0.32	NS	-3.74	0.047	-4.20	0.028	-1.95	0.039	-2.01	0.034
	(-1.18,0.72)		(-1.25,0.60)		(-7.40,-0.07)		(-7.93,-0.47)		(-3.81,-0.10)		(-3.86,-0.15)	
**HDL-C**												
**<1.04**	0 (ref.)		0 (ref.)		0 (ref.)		0 (ref.)		0 (ref.)		0 (ref.)	
**≥1.04**	1.36	NS	0.57	NS	0.01	NS	0.23	NS	0.63	NS	0.43	NS
	(-1.83,4.56)		(-2.72,3.86)		(-0.71,0.73)		(-0.48,0.94)		(-0.86,2.13)		(-1.10,1.95)	
**LDL-C**												
**<3.5**	0 (ref.)		0 (ref.)		0 (ref.)		0 (ref.)		0 (ref.)		0 (ref.)	
**≥3.5**	1.60	NS	2.96	NS	-0.03	NS	0.19	NS	0.80	NS	1.02	NS
	(-2.29,5.49)		(-1.09,7.02)		(-1.02,0.96)		(-0.78,1.17)		(-1.14,2.73)		(-0.92,2.97)	

NGT: normal glucose tolerance; IGR: impaired glucose regulation; DI: disposition index; TG: triglyceride; TC: total cholesterol; HDL-C: high-density lipoprotein cholesterol; LDL-C: low-density lipoprotein cholesterol.

*Analysis was conducted after adjusting for SBP, DBP, BMI, WHR.

## Discussion

The present study has shown that normal glucose tolerance individuals with dyslipidemia had severe insulin resistance and impaired β cell function. Within IGR groups, dyslipidemia might diminish the compensatory insulin secretion to insulin resistance in subjects with CGI. TG and HDL-C were correlated with insulin resistance, while TG and TC were negatively correlated with pancreatic β cell response to insulin resistance in individuals with NGT and prediabetes.

T2DM has become a major public health problem affecting populations at all levels of socioeconomic development in the world [12, 13]. Dyslipidemia is known as an independent risk factor for the development of T2DM [14]. Chen et al. [15] have found that subjects with hyperlipidemia were more than 3 times higher at risk of developing T2DM compared with participants with normal lipid. However, the influence of dyslipidemia in the process from NGT to diabetes is still obscure.

In the present study, we found that NGT individuals with dyslipidemia had higher HOMA-IR and lower DI levels, which indicated the existence of severe insulin resistance and impaired compensatory response of β cell to insulin resistance.

It is well known that the development of T2DM is characterized by the presence of insulin resistance and progressive decrease of β cell function that is not able to sufficiently compensate for glucose increase [16]. A previous study showed that the transition from NGT to IGR is associated with the deterioration of insulin sensitivity, but glucose tolerance in this process is only mildly impaired because of the compensatory increase in insulin secretion and resultant hyperinsulinemia [17]. However, blood insulin levels should not be equated with β cell function. In general, β cells respond to an incremental change in glucose with an incremental change in insulin, and this response moderated with the severity of insulin resistance. Therefore, disposition index (DI) [ΔI/ΔG ÷ IR] is sound to be more effective on β cell function evaluation. In addition, the DI has been regarded as an accurate assessment of β cell function in previous literature [18] and was used to investigate β cell function in many studies [19–21]. In this study, we observed individuals with NGT and dyslipidemia had a significant decline of DI level, which means dyslipidemic patients, even with normal glucose tolerance, have already showed impaired glucose metabolism. Thus, for individuals with dyslipidemia, appropriate preventions should be carried out to prevent the progression from dyslipidemia to prediabetes and diabetes.

To further analyze and compare the effect of dyslipidemia on individuals with IGR, we divided these subjects into three subgroups, i.e. IFG, IGT and CGI. It is noteworthy that isolated IFG and IGT have different pathophysiologies, which have been discussed and still under discussion. In some studies, insulin resistance was the primary abnormity of IGT, while IFG was more associated with insulin secretion [22, 23]. However, other investigators have reported the opposite results [24]. In Japanese and Korean, impaired early-phase insulin secretion plays more important role in isolated IGT, whereas insulin resistance is dominant in isolated IFG subjects [25, 26]. In the present study, we found individuals with CGI had the highest level of insulin resistance, while subjects with IGT had better insulin sensitivity than with IFG. These findings were consistent with results from Novoa et al. [27], which indicated CGI group had the highest levels of insulin resistance, and other studies have also shown that subjects with IFG have increased insulin resistance when compared with IGT group [28, 29].

However, in our study, no significant difference of insulin resistance was found among IFG, IGT and CGI groups with or without dyslipidemia. These may because that HOMA-IR was calculated with fasting plasma glucose (FPG) and fasting insulin (FINS). The CGI group had higher levels of fasting glucose and fasting insulin than IGT and IFG groups, and IFG group had higher level of fasting glucose than IGT group (data not shown). But no statistical differences of FPG and FINS were found among IFG, IGT, CGI groups with or without dyslipidemia. These results indicated that dyslipidemia might increase insulin resistance in normoglycemic individuals, while in individuals already have moderate to severe insulin resistance, dyslipidemia seems to have slight influence. A possible explanation is that longtime and severe insulin resistance could result in the decrease of insulin secretion, which may alleviate further deterioration of insulin resistance.

In the current study, we found that both IFG and CGI groups had lower levels of DI than IGT group, and the DI level in CGI subjects with dyslipidemia was significantly declined when compared to those with normal lipid level. These results suggest that IFG subjects have a more prominent defect in β cell function than IGT individuals, which were consistent with the findings from Hong et al. [30]. Moreover, dyslipidemia might injure β cell function of IGR groups, especially the CGI group.

Although it has been demonstrated that dyslipidemia plays a negative role in β cells functions of non-diabetic individuals, the concrete effects of different lipid profiles (TG, TC, HDL-C and LDL-C) on insulin sensitivity and β cell function are still unclear. After multiple linear regression analysis, we found that TG and HDL-C were correlated with insulin resistance, while TG and TC were negatively correlated with pancreatic β cell response to insulin resistance in non-diabetic individuals.

The relationship between high TG and/or low HDL-C levels and T2DM is well known and the American Diabetes Association recommends that adults of any age should be screened for diabetes mellitus if they have a TG levels >250 mg/dL and/or HDL-C levels <35 mg/dL[31]. Moreover, Giannini et al. [32] proposed that the TG to HDL-C ratio is correlated with insulin sensitivity and may be used to identify subjects at high risk of insulin resistance induced death. Several studies have shown that both hypertriglyceridemia and hypercholesteromia diminish glucose stimulated insulin secretion by inhibiting glucose oxidation via reducing pyruvate dehydrogenase (PDH) activity and elevating PDH kinase activity [33–36]. Moreover, increased TG or TC levels trigger β cell apoptosis by increasing the production of ceramide and nitric oxide [37–41]. Our data appear to provide further evidence that TG and TC were closely correlated with pancreatic β cell function in non-diabetic individuals.

Our study may have some limitations. First, although the euglycemic clamp and the intravenous glucose tolerance test are considered as the “gold standard” method to evaluate insulin resistance and pancreatic β cell function, these tests are expensive, time-consuming and not applicable in large epidemiological studies. So we used OGTT to instead of these tests. Second, the pathophysiological basis of impaired glucose regulation may vary in different ethnic study populations. Third, potential bias from daily diet or exercise may exist in the study.

## Conclusions

TG and HDL-C were correlated with insulin resistance, and TG, TC were negatively correlated with pancreatic β cell response to insulin resistance in non-diabetic individuals. Subjects with dyslipidemia and NGT have impaired insulin sensitivity and β cell function. Dyslipidemia might diminish pancreatic β cell function in subjects with CGI. Therefore, an early and “aggressive” intervention based on lifestyle changes and insulin sensitizer drugs in subjects with dyslipidemia seems to be recommended because of the high risk of developing T2DM.

## Supporting information

S1 File Clinical datasets. The datasets supporting the conclusions of this article.
(XLSX)
